# Prediction of lactate concentrations after cardiac surgery using machine learning and deep learning approaches

**DOI:** 10.3389/fmed.2023.1165912

**Published:** 2023-09-14

**Authors:** Yuta Kobayashi, Yu-Chung Peng, Evan Yu, Brian Bush, Youn-Hoa Jung, Zachary Murphy, Lee Goeddel, Glenn Whitman, Archana Venkataraman, Charles H. Brown

**Affiliations:** ^1^Johns Hopkins University, Baltimore, MD, United States; ^2^Department of Anesthesiology & Critical Care Medicine, Johns Hopkins University School of Medicine, Baltimore, MD, United States; ^3^Department of Anesthesiology & Critical Care Medicine, University of Michigan, Ann Arbor, MI, United States; ^4^Department of Surgery, Johns Hopkins University School of Medicine, Baltimore, MD, United States; ^5^Department of Electrical and Computer Engineering, Johns Hopkins University Whiting School of Engineering, Baltimore, MD, United States

**Keywords:** lactate, cardiac surgery, prediction, malperfusion, machine learning

## Abstract

**Background:**

Although conventional prediction models for surgical patients often ignore intraoperative time-series data, deep learning approaches are well-suited to incorporate time-varying and non-linear data with complex interactions. Blood lactate concentration is one important clinical marker that can reflect the adequacy of systemic perfusion during cardiac surgery. During cardiac surgery and cardiopulmonary bypass, minute-level data is available on key parameters that affect perfusion. The goal of this study was to use machine learning and deep learning approaches to predict maximum blood lactate concentrations after cardiac surgery. We hypothesized that models using minute-level intraoperative data as inputs would have the best predictive performance.

**Methods:**

Adults who underwent cardiac surgery with cardiopulmonary bypass were eligible. The primary outcome was maximum lactate concentration within 24 h postoperatively. We considered three classes of predictive models, using the performance metric of mean absolute error across testing folds: (1) static models using baseline preoperative variables, (2) augmentation of the static models with intraoperative statistics, and (3) a dynamic approach that integrates preoperative variables with intraoperative time series data.

**Results:**

2,187 patients were included. For three models that only used baseline characteristics (linear regression, random forest, artificial neural network) to predict maximum postoperative lactate concentration, the prediction error ranged from a median of 2.52 mmol/L (IQR 2.46, 2.56) to 2.58 mmol/L (IQR 2.54, 2.60). The inclusion of intraoperative summary statistics (including intraoperative lactate concentration) improved model performance, with the prediction error ranging from a median of 2.09 mmol/L (IQR 2.04, 2.14) to 2.12 mmol/L (IQR 2.06, 2.16). For two modelling approaches (recurrent neural network, transformer) that can utilize intraoperative time-series data, the lowest prediction error was obtained with a range of median 1.96 mmol/L (IQR 1.87, 2.05) to 1.97 mmol/L (IQR 1.92, 2.05). Intraoperative lactate concentration was the most important predictive feature based on Shapley additive values. Anemia and weight were also important predictors, but there was heterogeneity in the importance of other features.

**Conclusion:**

Postoperative lactate concentrations can be predicted using baseline and intraoperative data with moderate accuracy. These results reflect the value of intraoperative data in the prediction of clinically relevant outcomes to guide perioperative management.

## Introduction

A critical goal of intraoperative management is optimizing systemic perfusion to maintain organ function. During cardiac surgery, both hemodynamic changes inherent to the surgery and management strategies while on cardiopulmonary bypass can reduce systemic perfusion. However, impaired systemic perfusion may not be evident until hours to days after surgery.

Many prediction models in cardiac surgery use simple combinations of static patient characteristics and a limited number of intraoperative variables (1–3). However, during cardiac surgery, and particularly during cardiopulmonary bypass, minute-level data is available on key parameters that are thought to affect perfusion (e.g., flow, hemoglobin concentration, mean arterial pressure) or measure the adequacy of perfusion (e.g., acid/base status, mixed venous saturations). Although conventional prediction models often ignore this dynamic data, deep learning approaches are well-suited to incorporate time-varying and non-linear data with complex interactions (4).

There is no gold standard to measure the adequacy of overall systemic perfusion, but blood lactate concentration is one important biomarker in clinical care (5, 6). Hyperlactemia after cardiopulmonary bypass has been consistently associated with postoperative morbidity, increased duration of intensive care stay, and mortality (6–8). Immediate elevations in postoperative lactate at the time of intensive care unit admission are thought to be largely due to hypoperfusion and have the strongest associations with important postoperative outcomes, including longer duration of intensive care unit stay and in-hospital mortality (8, 9). However, elevated lactate concentrations up to 24 h after surgery have also been associated with worse in-hospital and long-term mortality, potentially implicating hypoperfusion as a risk factor (10). Thus, it is important to understand which patients may develop postoperative elevations in lactate, as well as what intraoperative factors are contributory.

The goal of this study was to use several machine learning and deep learning approaches to predict maximum blood lactate concentrations in patients up to 24 h after cardiac surgery. We hypothesized that the addition of intraoperative and cardiopulmonary bypass parameters to baseline patient and surgical characteristics would improve prediction models; consequently, models using minute-level intraoperative data that included multiple parameters related to cardiopulmonary bypass would have the best predictive performance. Further, we aimed to examine the relative importance of predictive features using Shapley additive values (SHAP values). Although the prediction models were designed to use all intraoperative data, the results would potentially support the need for real-time prediction models that could be used during surgery to guide management decisions that could improve systemic perfusion.

## Materials and methods

### Institutional review board and consent

This study was approved by the Johns Hopkins Institutional Review Board (Baltimore, MD) with waived patient consent. The Transparent Reporting of a multivariable prediction model for Individual Prognosis or Diagnosis (TRIPOD) framework was used to develop and report the results of this study (11).

### Study population

**Figure 1:**
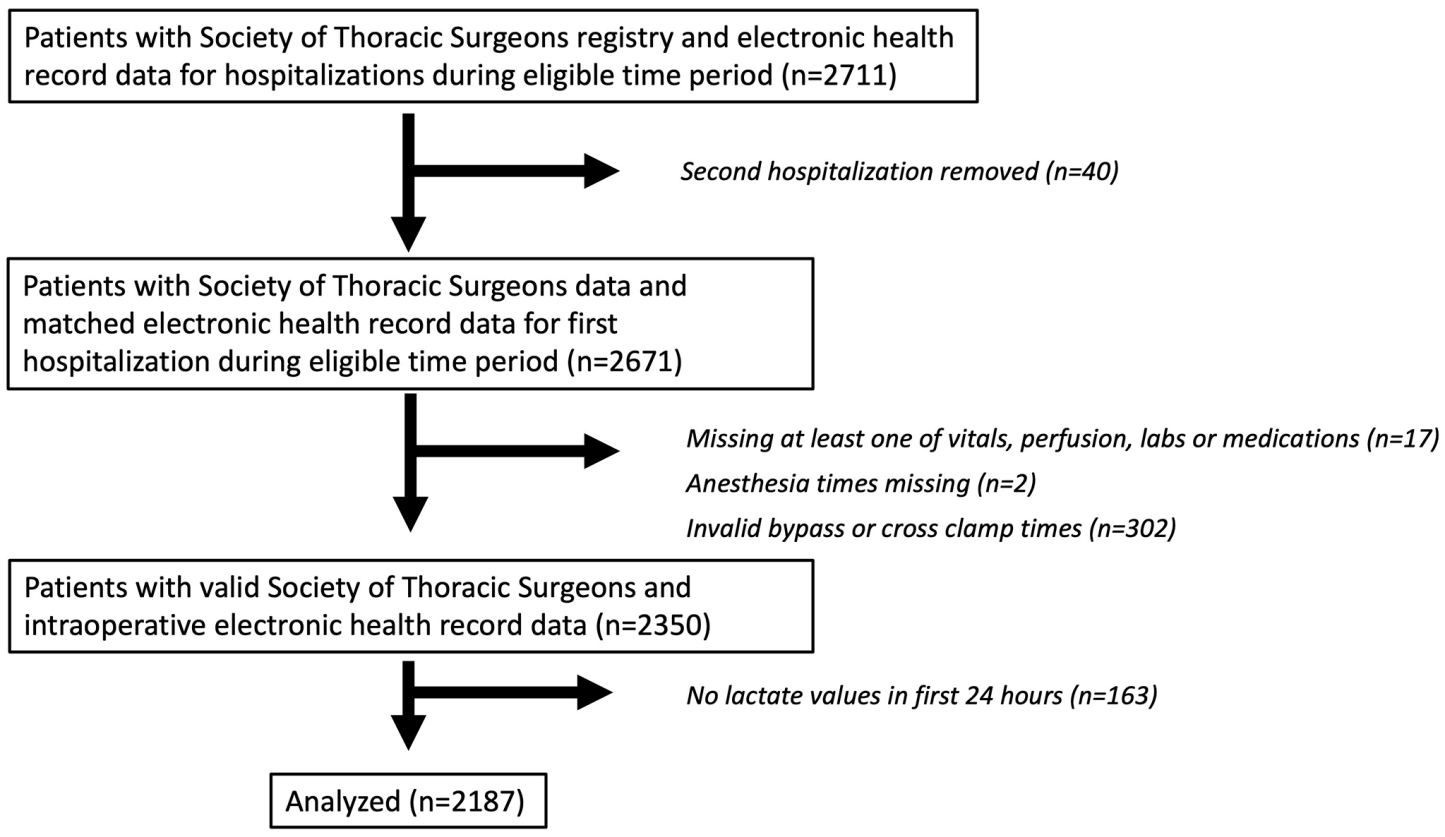
Patient flow diagram. A patient flow diagram is shown describing included and excluded patients in the analytic cohort.

Our study includes patients 18 years or older who underwent cardiac surgery with cardiopulmonary bypass at Johns Hopkins between July 1, 2016, and October 31, 2019. Patient data was extracted from Epic and merged with data from the Society of Thoracic Surgeons Registry. The Society of Thoracic Surgeons Registry captures patient preoperative risk characteristics, procedure-related processes of care, and clinical outcomes. The registry has been shown to have extremely high inter-rater reliability and completeness and is widely regarded as the “gold standard” for benchmarking cardiac surgery risk adjusted outcomes (12). For patients with more than one hospitalization with surgery during this time period, only the first hospitalization with surgery was included. Other exclusion criteria included missing time stamps and missing at least one recorded value of postoperative lactate within the first 24 h. Further filtering was done to account for errors in the information gathering stage, such as missing anesthesia start/end times. For patients with multiple surgeries during one hospitalization, the first surgery was predominantly used, but a second surgery which met criteria could also be eligible. These steps yielded a total of *N* = 2,187 patients for the analysis (Figure 1). The sample size for this analysis was derived using all available data that met inclusion criteria.

### Variable selection and data preprocessing

Variables from both the Society of Thoracic Surgeons Registry and from EPIC were reviewed by an expert clinician for potential inclusion in the prediction models based on clinical relevance and categorization as preoperative, intraoperative, or postoperative data. Static variables with greater than 5% missingness were excluded. A list of included variables is shown in Supplementary Table S1.

Data extracted from EPIC provided granular baseline and intraoperative variables. Continuous intraoperative data (e.g., pulse oximetry) was extracted at 1 min intervals, and other discrete data were extracted as time-stamped variables. Medications were considered as total intraoperative dose or maximum infusion rate. The following pre-processing steps were applied to static variables: (1) imputation of missing categorical/binary features (i.e., comorbidities and medications) to be the absent value of zero, and (2) removal of outliers via clipping of continuous features to 0.01 and 0.99 percentiles. For the intraoperative time series, outliers were identified and removed based on parameters set by clinical judgment. Missing continuous static values were imputed based on the population mean. Missingness in the time series was imputed based on the mean value in the analytic cohort. The pulse variable was set to 0 for the duration of cross-clamp. Only the pulse measurements outside of cross-clamp were considered when computing intraoperative summary statistics.

We considered intraoperative variables using two approaches. In the first approach, we summarized all intraoperative features (minute-level continuous intraoperative data, labs) as separate fixed size vectors, using the mean value and variance across the whole intraoperative window for which time series data was available. We also created several features based on time outside of clinically important ranges (mean arterial pressure <55, 60, and 65 mm Hg., central venous pressure >15 mm Hg., pulse >100, pulse <50, bicarbonate concentration <21 meq/L, hematocrit <21%, mixed venous saturation <70 and 75%, and pH <7.35). In the second approach, we utilized the dynamic information of the time-series data. To decrease noise in the data and reduce input data size, we compressed the data by taking the mean of every five minutes/measurements.

### Clinical end point

The primary outcome was the maximum lactate concentration recorded within the first 24 h after the end of anesthesia.

### Machine learning models

**Figure 2:**
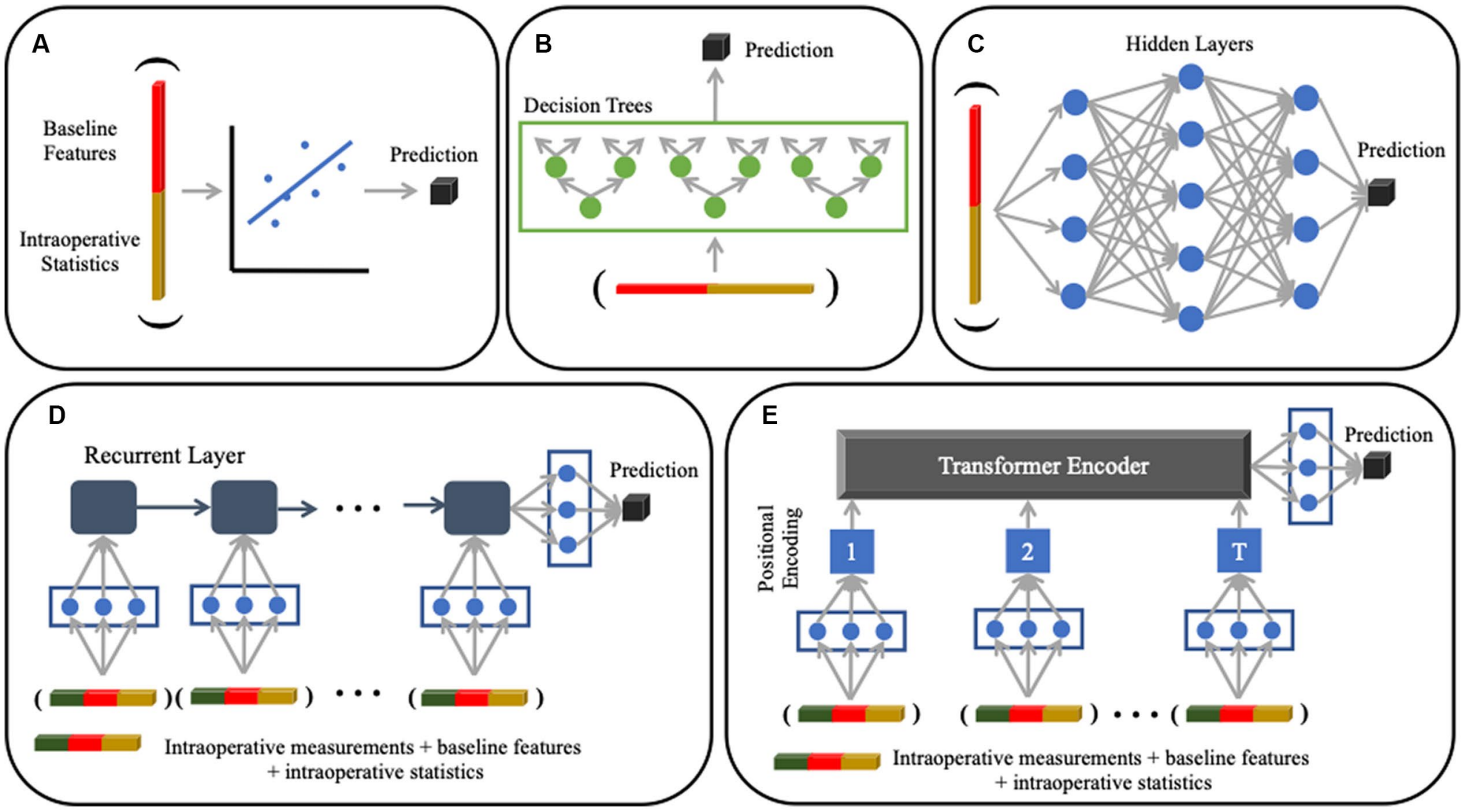
Machine learning models used for analysis. **(A)** Multivariate linear regression with an ElasticNet penalty (not pictured) to encourage feature sparsity. **(B)** Random Forest regression that uses an ensemble of decision tree classifiers for prediction; each tree is grown by iteratively searching for the feature that splits the patients into clusters with the lowest pairwise difference of lactate values within each cluster. **(C)** Artificial Neural Network that uses a sequence of fully connected layers to project the data onto latent representations (denoted by blue dots). The models in Panels **(A–C)** use static baseline and intraoperative variables as input variables. **(D)** Recurrent Neural Network that handles dynamic sequences through a series of feedback operations; this model can accommodate different input sequence lengths. **(E)** Transformer that uses a self-attention mechanism to model both short-and long-range dependencies in the input sequence. The models in **(D)** and **(E)** use time series intraoperative data, as well as static baseline and intraoperative variables, as input variables.

We conducted a comprehensive evaluation across five different machine learning models of increasing complexity: linear regression, random forest regression, artificial neural networks, recurrent neural networks, and transformers (Figure 2). The first three methods operate on static variables derived from both the preoperative data and intraoperative statistics. The last two methods can mine information directly from the intraoperative time series. Below, we provide brief summaries of each method, followed by our three evaluation paradigms in the next section.

**Linear regression (static)**: we use a multivariate linear model to predict the clinical endpoint (maximum postoperative lactate) based on the patient variables. Given the large number of input features, we use the ElasticNet model, which combines both L1 and L2 regularization on the learned regression coefficients. Mathematically, the regularization is done by adding the absolute value (L1) and squared value (L2) of each coefficient to the mean-squared error objective. Linear regression assumes a static input of fixed dimension. Hence, as noted above, we only apply it to the static preoperative variables and summarized intraoperative statistics.

**Random forest (static)**: the random forest is an ensemble of decision tree classifiers that incorporate two layers of randomness. Each tree is grown using a random subset of patients by recursively searching for the feature that splits the patients into two clusters with the lowest squared difference in lactate between all points assigned a cluster. This process is controlled via two hyperparameters, one governing the number of trees in the forest, and the other specifying the percentage of random features at each branch. The random forest requires a fixed input feature dimension and is only applied to static variables.

**Artificial neural network (static)**: we used a feedforward artificial neural network (ANN) to map the static input features onto the desired clinical outcome. The network consists of fully connected layers and ReLU activations after each layer. Tunable hyperparameters include the learning rate, hidden layer sizes, and the number of hidden layers. We use the Adam optimizer with the regularization parameter fixed to 1e-2 and a fixed dropout of 0.5 at each hidden layer.

**Recurrent neural network (static + time series)**: recurrent neural networks are extensions to the artificial neural network that can handle dynamic sequences of variable lengths. We relied on a gated recurrent unit architecture (13). The static features are concatenated to the dynamic features at every time point. Once again, we tuned the learning rate, hidden layer sizes, and the number of hidden layers. We use the Adam optimizer with the regularization parameter fixed to 1e-2 and a fixed dropout of 0.5 at each hidden layer and 0.05 at the input.

**Transformer (static + time series)**: transformers are a recently proposed alternative to recurrent architectures that use self-attention to learn representations of the dynamic inputs. We rely on the original encoder implementation by Vaswani et al. (14) and the extension to multivariate time series data by Zerveas et al. (15) tunable hyperparameters include the learning rate, hidden layer sizes, number of attention heads, and the dimension for the feedforward network model We use the Adam optimizer with the regularization parameter fixed to 1e-2 and a fixed dropout of 0.5 at each hidden layer and 0.05 at the input.

## Statistical analysis

We use a repeated nested five-fold cross validation to robustly quantify the performance of each model. Here, the dataset of *N* = 2,187 patients was randomly divided into five groups, i.e., folds. During each iteration, four folds were combined and split into training and validation. The validation set was used to determine the best hyperparameter configuration and set the early stopping criteria for each deep learning model. We use an automatic hyperparameter optimization framework called Optuna to set the hyperparameters of each method based on sampling 50 configurations via the tree-based Parzen Estimator. Once the hyperparameters were fixed, the models were optimized by minimizing the mean-squared error between the measured and predicted lactate values in the training dataset. We then evaluated the lactate prediction on the held-out testing fold. This nested procedure mitigates overfitting. We resampled the cross-validation folds five times to obtain performance confidence intervals for each model.

We used the SHapley Additive exPlanations (SHAP) approach as a model agnostic framework to identify the most important features for each model, and thus interpret the information being learned. SHAP values compute the change in the expected model prediction when conditioning on each feature of interest. Classic SHAP value estimation requires retraining a model on all feature subsets S⊆F , where F is the set of all features. To compute the importance of feature i, a model would be trained with and without the feature present. Predictions of the two models are subtracted, yielding the “effect” of feature i. This is computed for all subsets S , and computing a weighted average of these computed differences yields the SHAP value.

To reduce the computational overhead, we used the KernelSHAP method to jointly approximate the exact SHAP values. KernelSHAP solves a weighted linear regression with a specialized weighting kernel known as the Shapley kernel (16). KernelSHAP method provides better sample efficiency than the direct computation above. We extract variable importances by first running KernelSHAP on each testing fold in our 5-fold cross validation setup, and averaging the absolute SHAP values over the folds. This analysis is designed to align with how the models are trained and evaluated, and it provides insight on the behavior of the models used to generate the out-of-sample predictions in our main result.

## Results

### Patient characteristics

A total of 2,187 patients were included in this analysis, and a patient flow diagram is shown in Figure 1. Characteristics of patients are shown in Table 1. Mean age was 62 ± 13 years and 71% were male. Isolated coronary artery bypass graft surgery was performed in 50% of patients. Median duration of cardiopulmonary bypass was 102 min (Interquartile range [IQR] 75, 144). The maximum lactate concentration in the 24 h after surgery was a median of 4.6 mmol/L (IQR 2.8, 7.3). The median time to maximum postoperative lactate concentration from the end of the operation was 3.7 h (IQR 2.1, 5.8). Variables that were used as inputs to the models are listed in Supplementary Table S1 and include variables from both the Society of Thoracic Surgeons Registry and from the electronic medical record.

**Table 1:** Patient and perioperative characteristics.

	All Patients (*n* = 2,187)	Lactate >3 mmol/L (*n* = 1,550)	Lactate <=3 mmol/L (*n* = 637)	Value of *p*
Age (years), mean (SD)	62 (13)	62 (13)	60 (14)	<0.001^a^
Male, *n* (%)	1,558 (71.2%)	1,138 (73.4%)	420 (65.9%)	<0.001^b^
Race, *n* (%)				0.079^b^
Caucasian	1,517 (69.4%)	1,065 (68.7%)	452 (71.0%)	
Black	411 (18.8%)	279 (18.0%)	132 (20.7%)	
Asian	129 (5.9%)	99 (6.4%)	30 (4.7%)	
Other	113 (5.2%)	88 (5.7%)	25 (3.9%)	
Comorbidities, *n* (%)				
Prior Stroke	170 (7.8%)	116 (7.5%)	54 (8.5%)	0.484^b^
Hypertension	1,618 (74.0%)	1,154 (74.5%)	464 (72.8%)	0.468^b^
Chronic Lung Disease	220 (10.1%)	160 (10.3%)	60 (9.4%)	0.576^b^
Obstructive Sleep Apnea	353 (16.1%)	254 (16.4%)	99 (15.5%)	0.671^b^
Tobacco Use	364 (16.6%)	220 (14.2%)	144 (22.6%)	<0.001^b^
Diabetes	773 (35.3%)	535 (34.5%)	238 (37.4%)	0.224^b^
Surgery, *n* (%)				<0.001^b^
Coronary Artery Bypass Only	1,101 (50.3%)	754 (48.6%)	347 (54.5%)	
Valve Surgery Only	302 (13.8%)	190 (12.3%)	112 (17.6%)	
Coronary Artery Bypass + Valve Only	143 (6.5%)	122 (7.9%)	21 (3.3%)	
Aortic Procedures Only	77 (3.5%)	59 (3.8%)	18 (2.8%)	
Ventricular Assist device	51 (2.3%)	45 (2.9%)	6 (0.9%)	
Heart Transplant	32 (1.5%)	30 (1.9%)	2 (0.3%)	
Other	481 (21.7%)	350 (22.6%)	131 (20.6%)	
Status, *n* (%)				<0.001^b^
Elective	1,006 (46.0%)	686 (44.3%)	320 (50.2%)	
Urgent	1,012 (46.3%)	720 (46.5%)	292 (45.8%)	
Emergent	146 (6.7%)	122 (7.9%)	24 (3.8%)	
Cardiopulmonary bypass duration (min), median (IQR)	102 (75, 144)	109 (79, 153)	90 (67, 118)	<0.001^a^
Aortic cross-clamp duration (min), median (IQR)	69 (49, 96)	73 (52, 103)	61 (43, 85)	<0.001^a^
Mean arterial pressure (mm Hg.), (median, IQR)	73.5 (69.9, 77.4)	73.2 (69.5, 76.9)	74.4 (70.7, 78.7)	<0.001^a^
Central Venous Pressure (mm Hg.), median, (IQR)	9.1 (7.4, 11.1)	9.1 (7.5, 11.1)	9.1 (7.2, 11.0)	0.174^a^
Pulse (beats per minute), median (IQR)	72.9 (67.1, 80.5)	74.0 (68.0, 81.3)	70.4 (65.5, 77.2)	<0.001^a^
Right cerebral oximetry, median (IQR)	62.6 (55.0, 69.5)	63.1 (55.4, 70.0)	61.2 (53.7, 69.0)	0.03^a^
Left cerebral oximetry, median (IQR)	62.2 (54.1, 69.5)	62.6 (54.6, 70.0)	60.4 (52.6, 68.7)	0.018^a^
Blood Flow (derived from cardiopulmonary bypass monitor) during bypass (L/min), median (IQR)	4.94 (4.46, 5.34)	4.93 (4.44, 5.33)	4.95 (4.49, 5.35)	0.730^a^
Bicarbonate Concentration (derived from cardiopulmonary bypass monitor) during bypass mmol/L, median (IQR)	24.9 (23.8, 26.1)	24.8 (23.7, 26.0)	25.1 (24.0, 26.2)	0.001^a^
Hematocrit (derived from cardiopulmonary bypass monitor) during bypass (%), median (IQR)	27.4 (24.2, 30.7)	27.4 (24.3, 30.9)	27.4 (24.2, 30.5)	0.596^a^
Mixed Venous Oxygen Saturation (derived from cardiopulmonary bypass monitor) during bypass (%), median (IQR)	83.1 (79.7, 86.8)	83.1 (79.7 86.8)	83.1 (80.0, 86.6)	0.988^a^
pH (derived from cardiopulmonary bypass monitor), during bypass, median (IQR)	7.39 (7.37, 7.41)	7.39 (7.37, 7.41)	7.39 (7.37, 7.42)	<0.001^a^
Maximum epinephrine infusion (mcg/kg/min) median (IQR)	0.05 (0.03, 0.07)	0.05 (0.03, 0.08)	0.05 (0.03, 0.05)	<0.001^a^
Maximum norepinephrine infusion (mcg/kg/min), median (IQR)	0.00 (0.00, 0.05)	0.00 (0.00, 0.05)	0.00 (0.00, 0.05)	<0.001^a^
Phenylephrine bolus administration (mcg), median (IQR)	700 (200, 1850)	700 (200, 2000)	700 (225, 1,500)	0.791^a^
Red blood cell transfusion (mL), median (IQR)	0 (0, 300)	0 (0, 500)	0 (0, 300)	0.002^a^

SD, standard deviation; IQR, inter-quartile range; L, liters; mL, milliliters; mm Hg, millimeters of mercury.

a Wilcoxon Rank Sum test.

b Chi-Square test.

### Comparison of model performance for lactate prediction

#### Baseline characteristics as model inputs

**Figure 3:**
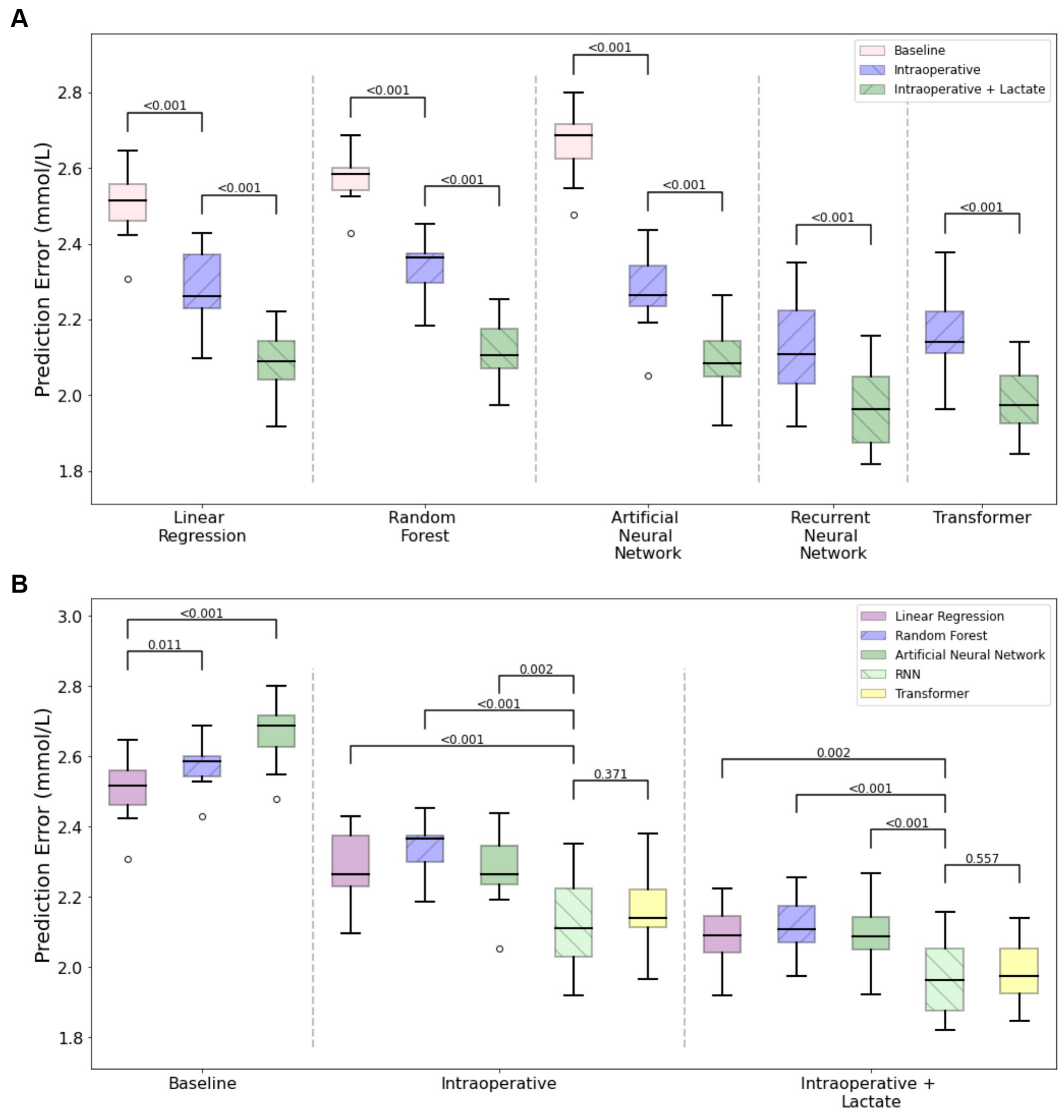
Prediction modelling for postoperative lactate concentration. Plots showing mean absolute error in predicting maximum lactate concentrations in the first 24 h after surgery. The results of five different machine learning models are shown, with separate models based on included variables (only baseline variables, baseline and intraoperative variables, baseline and intraoperative variables and all intraoperative lactate values). The linear regression, random forest, and artificial neural network models use summarized statistics of intraoperative data, while the recurrent neural network and transformer models incorporate time-series intraoperative data. Panel **(A)** demonstrates the additive value of more included variables while holding the model constant, with the feature set that includes baseline, intraoperative, and all intraoperative lactate values having the best model performance. Panel **(B)** is organized differently and demonstrates that the models that can utilize time series data have the best performance even when the type of data in the feature set is constant.

We first examined model performance (i.e., prediction error, expressed as the mean absolute error) for prediction of highest postoperative lactate using only baseline patient characteristics. As seen in Figure 3 and Table 2, for models that only used baseline characteristics as input variables, the mean prediction error of maximum postoperative lactate concentration ranged from a median of 2.52 mmol/L (IQRS 2.46, 2.56) for the linear regression model to a median of 2.58 mmol/L (IQRS 2.54, 2.60) for the random forest model.

**Table 2:** Model performance (mean absolute error^a^) for prediction of maximum lactate concentration in the 24 hours after cardiac surgery.

	Baseline patient characteristics	Intraoperative features (not including intraoperative lactate)	Intraoperative features (including intraoperative lactate)
Linear Regression (mean absolute error in mmol/L), median (IQR)	2.52 (2.46, 2.56)	2.26 (2.23, 2.37)^b^	2.09 (2.04, 2.14)^b^
Random Forest (mean absolute error in mmol/L), median (IQR)	2.58 (2.54, 2.60)	2.37 (2.30, 2.38)^b^	2.11 (2.07, 2.17)^b^
Artificial Neural Network (mean absolute error in mmol/L), median (IQR)	2.52 (2.49, 2.57)	2.28 (2.24, 2.37)^b^	2.12 (2.06, 2.16)^b^
Recurrent Neural Network (mean absolute error in mmol/L), median (IQR)	N/A	2.11 (2.03, 2.22)^c^	1.96 (1.87, 2.05)^c^
Transformer (mean absolute error in mmol/L), median (IQR)	N/A	2.14 (2.11, 2.22)^c^	1.97 (1.92, 2.05)^c^

a We computed the mean absolute error (MAE) for every testing fold to evaluate the out-of-sample generalization performance. We first used a single 5-fold cross validation to generate 5 MAE values. Then, we repeated the cross validation procedure three times by randomly shuffling the data. The result is a distribution of MAE values computed across the repeated testing folds (15 values in total). The entries in the table are reported as the median and interquartile intervals of this MAE distribution.

b Intraoperative features included static characteristics and distribution-based parameters for time-series data (e.g., mean, minimum, variance).

c Intraoperative features included minute-level time series data, in addition to static characteristics and distribution-based parameters for time-series data.

#### Baseline and static intraoperative characteristics as model inputs

We next included static intraoperative characteristics as input variables. Since not all institutions measure intraoperative lactate concentration during surgery, we examined the performance of each model with and without intraoperative lactate concentration as an input variable. For the three models that do not use time-series data (linear regression, random forest, and artificial neural network), the inclusion of all static intraoperative variables aside from lactate concentration improved model performance and reduced the mean prediction error to a range of median 2.26 mmol/L (IQR 2.23, 2.37) to median 2.37 mmol/L (IQR 2.30, 2.38). The inclusion of intraoperative lactate concentration further reduced prediction error to a range of median 2.09 mmol/L (IQR 2.04, 2.14) to median 2.12 mmol/L (IQR 2.06, 2.16) (Figure 3 and Table 2).

#### Baseline and time-series intraoperative characteristics as model inputs

Finally, the addition of intraoperative time-series data as inputs for recurrent neural network and transformer models resulted in the best model performance, with a reduction in prediction error of approximately 10%, compared to models that do not use time-series data. Without intraoperative lactate concentration as an input, the mean prediction error ranged from median 2.11 mmol/L (IQR 2.03, 2.22) to median 2.14 (IQR 2.11, 2.22). The inclusion of intraoperative lactate concentration further improved model performance and resulted in the lower prediction error of all models that were examined, with a mean prediction error that ranged from median 1.96 mmol/L (IQR 1.87, 2.05) to median 1.97 mmol/L (IQR 1.92, 2.05) (Figure 3 and Table 2).

Overall, the performance of each model consistently and significantly improved as more perioperative information (baseline, intraoperative, intraoperative + lactate data) was provided as input (Figure 3A). Additionally, the recurrent neural network model had significantly better performance than models which did not use time-series data, and even surpassed the transformer model with similar data inputs (Figure 3B). As *post hoc* sensitivity analyses, we also examined model performance in elective and non-elective surgeries, and found similar performance for the elective and urgent surgeries, although the prediction error was consistently lower in the small number of patients undergoing emergent surgery (Supplementary Table S2).

### Comparison of observed vs. predicted lactate values

**Figure 4:**
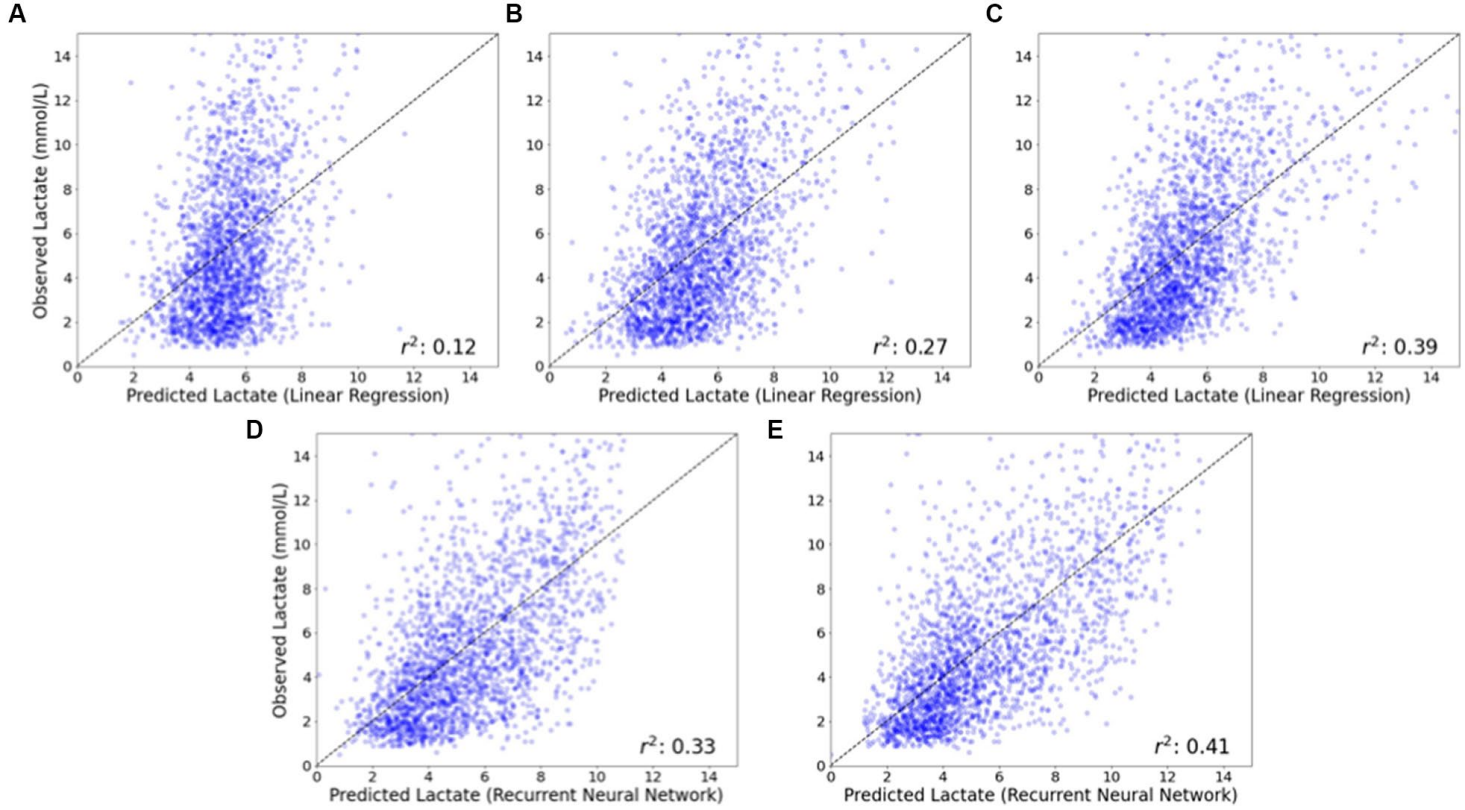
Observed vs. predicted postoperative lactate concentrations for linear regression and recurrent neural network models. The observed vs. predicted postoperative lactate concentration is shown for a representative model that does not utilize time-series data (linear regression) and a model that does utilize time-series data (recurrent neural network). For the linear regression model that does not utilize time-series data, the inclusion of intraoperative data **(B)**, and additionally intraoperative lactate concentration **(C)** substantially improves model performance compared to a model that only utilizes baseline patient data **(A)**. For the recurrent neural network model that does utilize time-series data, model performance is improved with the inclusion of intraoperative time series data **(D)** and additionally intraoperative lactate concentrations **(E)**, compared to models that do not utilize time-series data **(B)** and **(C)**.

Figure 4 depicts observed compared to predicted lactate values for two representative models: linear regression (which uses static variables as inputs) and recurrent neural network (which uses both static and time-series variables as inputs). Using only preoperative data as input variables resulted in the greatest error in observed to predicted values, while the addition of intraoperative data (and particularly time-series) data improved prediction. The scatterplots in Figure 4 demonstrate that prediction of maximum postoperative lactate values was better at lower levels of lactate (where points are closer to the diagonal) than at higher levels of lactate (where points are more distributed).

### Variable importance

**Figure 5:**
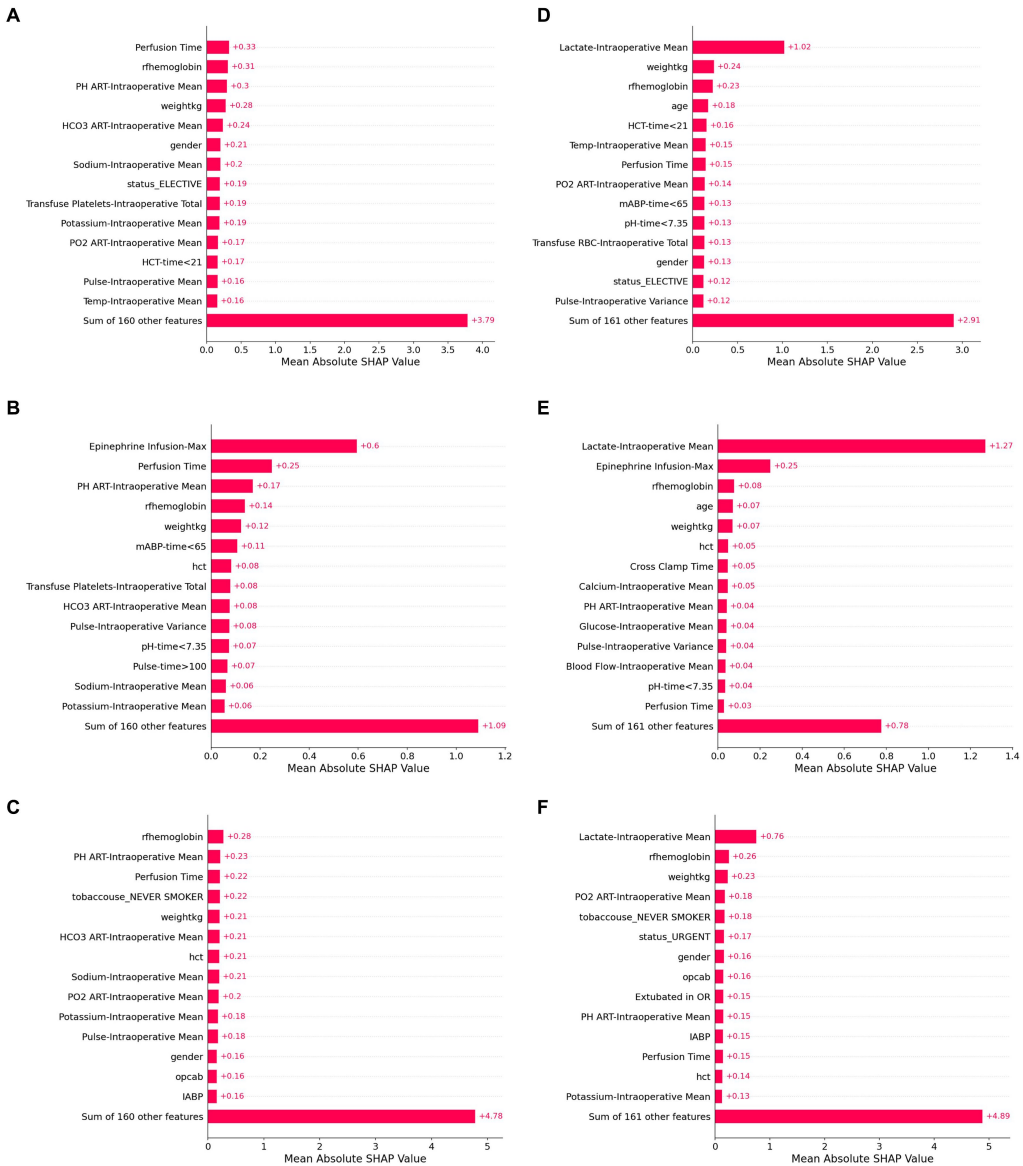
SHAP values for six model configurations to predict lactate concentration after cardiac surgery. Shapley additive values (SHAP values) represent an estimate of the contribution of each feature to the model prediction, with higher mean values indicating greater feature performance. The highest SHAP values are shown for linear regression **(A,D)**, random forest **(B,E)**, and artificial neural network models **(C,F)**. Panels **(A–C)** use baseline and intraoperative variables (but not intraoperative lactate) as inputs, while Panels **(D–F)** also include intraoperative lactate values as inputs.

The top fifteen SHAP values for linear regression, random forest and artificial neural network models are shown in Figure 5 and provide insight into the most important features for each prediction model. In all three models, intraoperative lactate concentration was the dominant feature. Baseline anemia and weight were also common important features (among the top five) for all three models as well. However, there was substantial heterogeneity in the remaining predictive features between models. As an example, for models that did not include intraoperative lactate concentration as an input, total dose of epinephrine infusion was the most important predictive feature in the random forest model but was not a top fifteen feature in either the linear regression or artificial neural network models.

When features were grouped to explore feature sets that had predictive power, we found that the contribution of intraoperative variables was higher in patients with the highest predicted postoperative lactate concentrations. On the other hand, for some patients with false prediction of high lactate, the intraoperative lactate values appeared to contribute substantial weight to the model, which may reflect that distribution-based approximations of time series variables in these models do not capture the dynamic course of perioperative physiologic changes.

## Discussion

The results of this study demonstrate that maximum lactate concentration in the 24 h after cardiac surgery can be predicted with moderate accuracy and a mean prediction error of 1.96 mmol/L for the best model (recurrent neural network). Compared to models with only static baseline and intraoperative patient characteristics, the inclusion of time-series intraoperative data generally improved model performance. The strongest and most consistent predictive feature was intraoperative blood lactate concentration. Other consistent predictive features included baseline anemia and weight. However, beyond these features, there was substantial heterogeneity in the strength of individual variables that informed model predictions.

Our results demonstrate that incorporation of intraoperative variables, and particularly time-series data, substantially improved model performance for the prediction of postoperative lactate concentration, as compared to models that only used baseline characteristics. Other prediction models for a variety of postoperative morbidity or mortality that have been developed using baseline and intraoperative data have demonstrated little to no improvement in predictive power with the inclusion of intraoperative data (17–19), while the results of our study demonstrate substantial improvement in predictive power with the use of intraoperative time-series data. In comparing the various modelling approaches, we found similar performance using linear regression, random forest, and artificial neural network approaches, each of which utilizes static variables (e.g., patient comorbidities) or distribution-based categorization of time-series variables (e.g., mean or maximum values). Deep learning approaches that utilized full time-series data (recurrent neural network and transformer) had the best performance, most notably when intraoperative lactate was included as a feature. There are several potential explanations for error in model predictions, including potentially important unmeasured factors, limitation in information that can be extracted from measured factors, and a heterogeneous cohort. However, these results imply that valuable information may be captured in the evolving dynamics of time series data, that reflect physiologic changes during surgery.

The best model performance which used baseline and intraoperative time-series variables achieved a mean prediction error of 1.96 mmol/L (IQR 1.87–2.05) for maximum postoperative lactate concentration. Increased blood lactate concentrations during and after surgery are thought to be due to an imbalance between tissue oxygen supply and demand (Type A lactic acidosis) or due to other non-hypoxic causes, such as glycolysis, drug therapy or hypothermia (Type B) (20). Although the exact contribution of each etiology to blood lactate concentrations cannot be known in individual patients, increased blood lactate early after cardiac surgery is highly clinically relevant and has been consistently associated with postoperative morbidity and mortality (6, 7). There does appear to be greater importance to early elevated lactate concentration compared to late elevated lactate concentration with respect to mortality (8). However, elevated lactate concentrations up to 24 h after cardiac surgery have been associated with in-hospital and long-term mortality (10). Thus, timely prediction of postoperative lactate concentrations may help risk stratify patients and guide ongoing resuscitation during and immediately after surgery, especially for those patients at high risk of developing elevated postoperative lactate concentrations. An important implication of our study is that these results support future efforts to develop real-time prediction models that can be used to guide intraoperative management decisions. There are several modifiable factors during cardiac surgery that may affect systemic perfusion and can be modified in patients with predicted high lactate concentrations. Both cardiac output and hemoglobin concentration are highly modifiable and may be increased to increase systemic perfusion. Other important approaches include optimization of acid–base status, improving right and left ventricle function, and consideration of options for mechanical circulatory support, such as an intra-aortic balloon pump.

There are few studies that have developed models to predict lactate concentrations after cardiac surgery, in part because lactate is not routinely collected postoperatively. One study of >13,000 patients developed nomograms to predict elevated lactate after cardiac surgery, with AUCs of 0.799 (21). However, the classification task was simply predicting a postoperative lactate >4 mmol/L, which is an easier prediction problem and provides less information than predicting the highest postoperative lactate. Indeed, elevated lactate concentrations have been associated with postoperative mortality in a dose-dependent manner (7). Additionally, a limited number of intraoperative variables were used, and no time-series data was included. Thus, the relative contribution of intraoperative variables, including time-series variables, to the prediction has not been clear from prior studies.

Shapley additive values can provide insight into the contribution of individual features to model prediction. In our study, intraoperative lactate was by far the most predictive feature in all models. This may be due to the delayed sequelae of intraoperative events or to ongoing pathophysiology that promotes lactate release. Additionally, baseline anemia and weight were common important features. However, beyond these features, there was substantial heterogeneity in top predictive features between models. This observation highlights a limitation of using Shapley values to assign importance and/or causation to individual features. Heterogeneity in the importance of features identified in Shapley plots may be caused by many factors, including feature redundancy and collinearity, individual implementation choices, and an outcome that may not be related to measured features. Furthermore, there were features in our dataset which represented similar physiologic characteristics, and correlations of features may have led to bias in attribution of individual feature contribution in the Shapley methodology. Taken as a whole, these results identify potential limitations in interpreting individual characteristics from perioperative data derived from Shapley values.

Strengths of this study include a large cohort with prediction of a clinically relevant outcome using several different machine and deep learning approaches. However, there are several limitations. First, prediction approaches used all intraoperative data, and thus prediction information is not available to guide intraoperative management. A next step would be developing real-time updating models for use during surgery. Second, we used cross-fold validation, but using data from only one institution, and an important next step will be to validate these prediction models in other settings. Third, a prediction model is limited by the available data, and for this reason we used a comprehensive set of patient and intraoperative characteristics at 1 min resolution. However, as demonstrated in our results, increasing the amount of input data tends to improve performance. Thus, we believe that amassing large and diverse datasets remains an open problem in this space that is important for future contributions. Fourth, the outcome was highest lactate within 24 h after surgery, given associations with in-hospital and long-term mortality. Others have reported a benign course for postoperative lactate elevations in the 6–12 h after cardiac surgery. Regardless of the timing, our results suggest that mechanisms that contribute to intraoperative elevations in lactate are also important for postoperative lactate elevations. Finally, the prediction of the best-performing model was moderate, but even with this degree of error, the prediction is still clinically relevant.

Postoperative lactate concentrations can be predicted using baseline and intraoperative data with moderate accuracy. These results support the need for real-time prediction models that could be used during surgery to guide management decisions that could improve systemic perfusion.

## Data availability statement

The data analyzed in this study is subject to the following licenses/restrictions: the datasets presented in this article are not available for immediate access because of potential for identification of patients. Requests to access the datasets should be directed to GW and will be considered with appropriate institutional agreements. Requests to access these datasets should be directed to gwhitman@jhmi.edu.

## Ethics statement

The studies involving humans were approved by Johns Hopkins Institutional Review Board. The studies were conducted in accordance with the local legislation and institutional requirements. The ethics committee/institutional review board waived the requirement of written informed consent for participation from the participants or the participants' legal guardians/next of kin because the data was retrospective and was collected for clinical purposes and met the criteria for usage in a study with waived consent.

## Author contributions

YK, Y-CP, and EY participated in data analysis, interpretation of the data, revising the manuscript critically for important intellectual content, approving the final version, and agrees to be accountable for all aspects of the work in ensuring that questions related to the accuracy or integrity of any part of the work are appropriately investigated and resolved. BB and Y-HJ participated in study design, data manipulation, interpretation of the data, revising the manuscript critically for important intellectual content, approving the final version, and agrees to be accountable for all aspects of the work in ensuring that questions related to the accuracy or integrity of any part of the work are appropriately investigated and resolved. ZM, LG, and GW participated in interpretation of the data, revising the manuscript critically for important intellectual content, approving the final version, and agrees to be accountable for all aspects of the work in ensuring that questions related to the accuracy or integrity of any part of the work are appropriately investigated and resolved. CB participated in study design, interpretation of the data, drafting the manuscript, approving the final version, and agrees to be accountable for all aspects of the work in ensuring that questions related to the accuracy or integrity of any part of the work are appropriately investigated and resolved. AV participated in study design, data analysis, interpretation of the data, revising the manuscript critically for important intellectual content, approving the final version, and agrees to be accountable for all aspects of the work in ensuring that questions related to the accuracy or integrity of any part of the work are appropriately investigated and resolved. All authors contributed to the article and approved the submitted version.

## Funding

This work was funded by a Malone Center for Engineering in Healthcare Seed Grant to AV and CB and K76AG057020 to CB.

## Conflict of interest

CB has received grant support from Medtronic in unrelated areas and discloses this relationship for all disclosures, regardless of whether or not it could be construed as a conflict. GW is the director of the executive study board for the Cryptics study, sponsored by Avania, and is the co-founder and co-owner of a start-up company to build a device for easier central line insertion (GWBN, LLC).

The remaining authors declare that the research was conducted in the absence of any commercial or financial relationships that could be construed as a potential conflict of interest.

## Publisher’s note

All claims expressed in this article are solely those of the authors and do not necessarily represent those of their affiliated organizations, or those of the publisher, the editors and the reviewers. Any product that may be evaluated in this article, or claim that may be made by its manufacturer, is not guaranteed or endorsed by the publisher.
