# Supplementary material for: Prediction of lactate concentrations after cardiac surgery using machine learning and deep learning approaches
Source: Front Med (Lausanne). 2023 Sep 14;10:1165912. doi: 10.3389/fmed.2023.1165912 (PMC10543087; doi:10.3389/fmed.2023.1165912)
Supplement: Supplementary file 1 [file Table_1.docx]

**Supplement Table 1: Features Utilized in Prediction Models**

| **Preoperative Features ^a^** | **Intraoperative Features** |
| --- | --- |
| Age | Static Features |
| Height (cm) | Circulatory Arrest ^i^ |
| Weight (kg) | Intra-Aortic Balloon Pump ^i^ |
| White Blood Cell Count | Extracorporeal Membrane Oxygenation ^i^ |
| Hemoglobin | Extubated in OR ^i^ |
| Hematocrit | Cardiopulmonary Bypass Time |
| Platelets | Cross Clamp Time |
| Last Creatinine Level | Time Series Features ^j^ |
| INR | Central Venous Pressure |
| Gender | Mean Arterial Blood Pressure |
| Race - White | Pulse |
| Race - Black / African American | Right cerebral oximetry |
| Race - Asian | Left cerebral oximetry |
| Race - American Indian / Alaskan Native | Blood Flow (derived from cardiopulmonary bypass monitor) |
| Race - Other | Bicarbonate Concentration (derived from cardiopulmonary bypass monitor) |
| Hispanic or Latino or Spanish Ethnicity | Hematocrit (derived from cardiopulmonary bypass monitor) |
| Family History of Premature Coronary Artery Disease | Mixed Venous Oxygen Saturation (derived from cardiopulmonary bypass monitor) |
| Diabetes | Temperature (derived from cardiopulmonary bypass monitor) |
| Dyslipidemia | pH (derived from cardiopulmonary bypass monitor) |
| Renal Failure-Dialysis | Calcium Ion Whole Blood |
| Hypertension | Glucose Whole Blood |
| Endocarditis | Hemoglobin Whole Blood |
| Chronic Lung Disease ^b^ | Lactate Whole Blood |
| Tobacco Use ^c^ | Potassium Whole Blood |
| Pulmonary Function Test ^d^ | Sodium Whole Blood |
| Arterial Blood Gas ^d^ | Bicarbonate Concentration (Arterial Blood Gas) |
| Home Oxygen | CO2 (Arterial Blood Gas) |
| Inhaled Medication or Oral Bronchodilator Therapy | O2 (Arterial Blood Gas) |
| Sleep Apnea | pH (Arterial Blood Gas) |
| Pneumonia | Urine |
| Illicit Drug Use | Epinephrine infusion |
| Depression | Epinephrine administration |
| Liver Disease | Norepinephrine infusion |
| Immunocompromise | Phenylephrine administration |
| Mediastinal Radiation | Transfusion of Red Blood Cells (ml) |
| Cancer Within 5 Years | Transfusion of Plasma (ml) |
| Peripheral Arterial Disease | Transfusion of Platelets (ml) |
| Thoracic Aorta Disease | Transfusion of Leukoreduced Red Blood Cells (ml) |
| Syncope |  |
| Unresponsive Neurologic State ^e^ |  |
| Cerebrovascular Disease |  |
| Prior Cerebrovascular Accident |  |
| Prior Transient Ischemic Attack |  |
| Previous Coronary Artery Bypass Surgery |  |
| Previous Valve Surgery |  |
| Heart Failure |  |
| Cardiogenic Shock |  |
| Resuscitation ^f^ |  |
| Meds-ACE Inhibitors or ARB Within 48 Hours |  |
| Meds-Amiodarone Prior to Surgery |  |
| Meds-Beta Blockers Within 24 Hours |  |
| Meds-Beta Blocker Therapy For More Than 2 Weeks Prior To Surgery |  |
| Meds-Calcium Channel Blocker Therapy For More Than 2 Weeks Prior To Surgery |  |
| Meds-Long-Acting Nitrate Therapy For More Than 2 Weeks Prior To Surgery |  |
| Meds-Nitrates-I.V. Within 24 Hours |  |
| Meds-Other Antianginal Medication Therapy For More Than 2 Weeks Prior To Surgery |  |
| Meds-ADP Inhibitors Within Five Days |  |
| Meds-Aspirin Within Five Days |  |
| Meds-Glycoprotein IIb/IIIa Inhibitor Within 24 Hours |  |
| Meds-Anticoagulants Within 48 Hours |  |
| Meds-Thrombolytics Within 48 Hours |  |
| Meds-Inotropes Within 48 Hours |  |
| Meds-Lipid Lowering Within 24 Hours |  |
| Meds-Steroids Within 24 Hours |  |
| Cardiac Catheterization Performed |  |
| Status (Elective, Urgent, Emergent) |  |
| Coronary Artery Bypass Surgery |  |
| Valve |  |
| Aorta Procedure Performed |  |
| Other Cardiac Procedure ^g^ |  |
| Atrial Fibrillation Procedure Performed |  |
| Other Non Cardiac Procedure ^h^ |  |
| Patient Admitted With Ventricular Assist Device |  |
| Ventricular Assist Device Implanted During This Hospitalization |  |
| Other Cardiac Procedure-ASD Repair - PFO Type |  |
| Other Cardiac Procedure -ASD Repair - Secundum Or Sinus Venosus |  |
| Other Cardiac Procedure -Atrial Fibrillation Intracardiac Lesions |  |
| Other Cardiac Procedure -Atrial Fibrillation Epicardial Lesions |  |
| Other Cardiac Procedure -Atrial Appendage Procedure |  |
| Other Cardiac Procedure -Arrhythmia Device Surgery |  |
| Other Cardiac Procedure -Lead Insertion |  |
| Other Cardiac Procedure -Arrhythmia Correction Surgery-Lead Extraction |  |
| Other Cardiac Procedure -Congenital |  |
| Other Cardiac Procedure -LV aneurysm |  |
| Other Cardiac Procedure -Subaortic Stenosis Resection |  |
| Other Cardiac Procedure -Surgical Ventricular Restoration |  |
| Other Cardiac Procedure -Transmyocardial Laser Revascularization |  |
| Other Cardiac Procedure -Card Transplant |  |
| Other Cardiac Procedure -Cardiac Trauma |  |
| Other Cardiac Procedure -VSD |  |
| Other Cardiac Procedure -Other |  |
| Prior Aortic Intervention |  |

^a^ All features are abstracted from the Society for Thoracic Surgeons Registry, which has precise definitions for each variable. ^b^ A feature reflecting presence and severity of disease. ^c^ Current or previous tobacco use. ^d^ A features reflecting whether or not the test was performed prior to surgery. ^e^ History of unresponsive state within 24 hours of time of surgery. ^f^ Cardiopulmonary resuscitation prior to operative procedure. ^g^ A variable indicating whether another cardiac procedure was performed other than CABG and valve. ^h^ A variable indicating whether a non-cardiac procedure was performed. ^i^ Features are defined as the presence or absence.  ^j^ Each feature was considered as a time-stamped value recorded at 1-minute intervals during the operation.

**Supplement Table 2: Model Performance** ^a^ **for Prediction of Maximum Lactate Concentration in the 24 Hours after Cardiac Surgery in Elective, Urgent, and Emergent Surgery**

| Data Source | Model | Elective | Urgent | Emergent |
| --- | --- | --- | --- | --- |
| Baseline Patient Characteristics | Linear Regression (absolute error in mmol/L), mean (std) | 2.543 (2.158) | 2.517 (2.183) | 2.169 (1.716) |
|  | Random Forest (absolute error in mmol/L), mean (std) | 2.582 (2.141) | 2.62 (2.154) | 2.237 (1.734) |
|  | Artificial Neural Network (absolute error in mmol/L), mean (std) | 2.555 (2.233) | 2.548 (2.231) | 2.2 (1.749) |
| Intraoperative Features (not including lactate) | Linear Regression (absolute error in mmol/L), mean (std) | 2.281 (1.942) | 2.332 (1.971) | 1.969 (1.561) |
|  | Random Forest (absolute error in mmol/L), mean (std) | 2.33 (1.923) | 2.412 (2.007) | 2.061 (1.573) |
|  | Artificial Neural Network (absolute error in mmol/L), mean (std) | 2.285 (2.041) | 2.338 (2.012) | 1.868 (1.563) |
|  | Recurrent Neural Network (absolute error in mmol/L), mean (std) | 2.157 (1.931) | 2.146 (1.969) | 1.778 (1.395) |
|  | Transformer (absolute error in mmol/L), mean (std) | 2.21 (1.878) | 2.189 (1.903) | 1.794 (1.446) |
| Intraoperative Features (including lactate) | Linear Regression (absolute error in mmol/L), mean (std) | 2.072 (1.724) | 2.121 (1.855) | 1.852 (1.515) |
|  | Random Forest (absolute error in mmol/L), mean (std) | 2.093 (1.793) | 2.163 (1.904) | 1.876 (1.547) |
|  | Artificial Neural Network (absolute error in mmol/L), mean (std) | 2.097 (1.843) | 2.167 (1.908) | 1.726 (1.565) |
|  | Recurrent Neural Network (absolute error in mmol/L), mean (std) | 1.998 (1.81) | 2.011 (1.877) | 1.668 (1.449) |
|  | Transformer (absolute error in mmol/L), mean (std) | 2.036 (1.784) | 2.012 (1.712) | 1.712 (1.451) |

^a^ The model performance is reported as the mean and standard deviation of the absolute error from one iteration of cross validation.
